# Effect of humic acid on oxidative stress and neuroprotection in traumatic spinal cord injury: an experimental study

**DOI:** 10.55730/1300-0144.5765

**Published:** 2021-07-10

**Authors:** Burak KINALI, Nail ÖZDEMİR, Ali KARADAĞ, Özge KAYA KORKMAZ, Ayşe Gülden DİNİZ, Fatma Demet ARSLAN

**Affiliations:** 1 Department of Neurosurgery, Medicana Bahçelievler Hospital, İstanbul, Turkiye; 2 Brain and Nerves, Neurosurgical Clinic, İzmir, Turkiye; 3 Department of Neurosurgery, Tepecik Research and Training Hospital, University of Health Sciences, İzmir, Turkiye; 4 Department of Pathology, Tepecik Research and Training Hospital, University of Health Sciences, İzmir, Turkiye; 5 Department of Pathology, Democracy University, İzmir, Turkiye; 6 Department of Biochemistry, Tepecik Research and Training Hospital, University of Health Sciences, İzmir, Turkiye

**Keywords:** Humic acid, spinal cord, trauma, oxidative stress, inflammation

## Abstract

**Background/aim:**

Traumatic spinal cord injury (TSCI) is an important health problem, especially in developing countries with additional socioeconomic loss. Humic acid (HA) usually has antioxidant, antiinflammatory, blood circulating, and antiviral effects. Hence, it was aimed herein to show the effect of HA on neuroprotection in a TSCI model.

**Materials and method:**

A TSCI model was used, in which 24 Wistar albino rats were divided into 4 groups: control group: subjected to only laminectomy; sham group: subjected to laminectomy + TSCI; HA 5 mg/kg group: subjected to laminectomy + TSCI + intraperitoneal (IP) injection of 5 mg/kg of HA; and HA 10 mg/kg group: subjected to laminectomy + TSCI + IP injection of 10 mg/kg of HA. Intracardiac blood samples were obtained preoperatively (preop), and at 1 and 24 h postoperatively (postop). The total antioxidant status (TAS), total oxidant status (TOS) and oxidative stress index (OSI) levels were evaluated in the serum. The motor functions were evaluated using the Modified Tarlov Score at 24 h postop.

**Results:**

There were no significant changes in the TAS values between the sham and HA 5 mg/kg and HA 10 mg/kg groups (p = 0.77/0.21). However there was a significant decrease in the TOS values at 24 h postop when comparing the sham and HA 5 mg/kg groups (p = 0.02). The pathological evaluation showed a significant decrease in the severity of edema, hemorrhage, polymorphonuclear leucocyte (PNL) infiltration, and mononuclear leucocyte (MNL)/macrophage/microglia infiltration when compared with the control group (p < 0.05). There was a significant recovery at the paraplegia level when the HA 5 mg/kg and HA 10 mg/kg groups were compared with the control group (p < 0.001).

**Conclusion:**

The effects of HA in the early stages of TSCI on oxidative stress, histopathological changes, and neurological improvement were investigated herein. It is thought to be a potential therapeutic agent in acute TSCI but needs to be further evaluated to determine the extent of its effect on other neuroprotective pathways in larger series.

## 1. Introduction

Traumatic spinal cord injury (TSCI) is an irreversible problem with an increasing number of occurrences. The world-wide frequency of TSCI is approximately 3.6–195.4/million [1], and there remains no consensus about effective medical treatment options for it. Despite the current treatment modalities, patients may need lifelong care and suffer serious physical and moral loss. This is a health problem that negatively affects both families and the economy of the countries they live in. In addition to the less studied active agents such as naloxone, thyrotropin-releasing hormone, and tirilazad, there are frequently used agents, such as monosialotetrahexosylganglioside (GM-1) (Sygen) and methylprednisolone, which are effective at various levels. None of these agents constitute treatment protocols based on level 1 and 2 evidence [2]. Moreover, various active agents have been studied in experimental TSCI models, such as glutamate receptor and ion channel antagonists, cyclooxygenase inhibitors, and erythropoietin. These agents are expected to be supported by further studies [3].

Humic acid (HA) is a polyphenolic substance that is used effectively in animal breeding and farming, and agriculture. HA contains various groups, such as phenol, carboxyl acid, and quinone groups, which change its effect. The characteristics of HA may vary depending on the source, age, climate, and environmental factors [4]. Today, it is known that HA accelerates and supports plant growth, and acts as a bactericidal in soil and fungicidal in plants [5]. In various studies, it has also been shown to be effective against pollution in soil and water and to have antiinflammatory, antibacterial, antiulcerogenic, and antiallergic features [6].

To date, the efficacy of HA in acute TSCI has not been shown in the literature. Therefore, it was aimed herein to investigate the efficacy of HA, which has not previously been studied in TSCI, by determining its antioxidant and oxidant effects and comparing these effects on histopathological and neurological outcomes.

## 2. Material and methods

Ethical approval for the study was obtained from the Ege University Animal Experiments Local Ethics Committee in İzmir (Approval number 2017-113). The study was conducted between August and September 2018. The first step of the study was performed at the Ege University Laboratory Animal Research and Application Center. Later, the biochemical evaluations were conducted at the Biochemistry Department of Health Sciences University, Tepecik, Izmir. The last step, the pathological evaluations, was conducted by the Pathology Department of the University of Health Sciences, Tepecik, Izmir. The study was conducted in accordance with the Experimental Animals Local Committee guidelines.

There were 28 Wistar-Albino rats in total, with an equal number of males and females among each group. They were between 8 and 12 weeks old and weighed 250–450 g. The rats were equally divided into 4 groups: laminectomy only (control), laminectomy + TSCI (sham), laminectomy + TSCI + low-dose HA (HA 5 mg/kg group), and laminectomy + TSCI + high-dose HA (HA 10 mg/kg group). Reference for the adaption of the HA doses was taken from the study of Ozkan et al. [7], but another low-dose HA 5 (mg/kg) group was also added. The rats were placed in separate cages at an optimal temperature of 18–21° C with an equal light/dark cycle and ad libitum food and water during the follow-up.

### 2.1. Experimental Protocol

The study groups were as follows:

Control group (n = 7): Only laminectomy (at T8–T10 level) without additional TSCI or medical therapy.
Sham group (n = 7): Laminectomy followed by TSCI (at T8–T10) and intraperitoneal (IP) injection of physiological saline.
HA 5 mg/kg (n = 7): Laminectomy followed by TSCI (at T8–T10) and IP injection of 5 mg/kg of HA.
HA 10 mg/kg (n = 7): Laminectomy followed TSCI (at T8–T10) and IP injection of mg/kg of HA.

From each group, 0.75 mm of intracardiac blood sample was aspirated 3 times: preoperatively (preop), and at 1 and 24 h postoperatively (postop). The HA injection was performed immediately after TSCI in the HA 5 mg/kg and HA 10 mg/kg groups. To euthanize the rats, thoracotomy was performed under high-dose anesthesia. The aorta was cannulated through the left ventricle and the descending aorta was clamped. The vascular system was perfused with 10% formaldehyde–phosphate-buffered saline (PBS). After perfusion, the spinal cord was dissected, and samples were taken to determine the level of laminectomy.

### 2.2. Operative procedure

The rats were anesthetized via IP injection of xylazine hydrochloride (10 mg/kg) (Rompon 2%; Bayer Health Care AG, Leverkusen, Germany) and ketamine hydrochloride (90–100 mg/kg) (Ketalar; Pfizer, New York, NY, USA). Moreover, 15 g/kg of prophylactic cefazolin sodium was subcutaneously injected at 1 h preop. In prone position, the dorsal region was cleared with povidone-iodine after shaving. Under a microscope, following the dorsal midline skin incision, the paravertebral muscles were dissected laterally, and laminectomy was performed at the level of T8–T10. The spinal cord was detected under the microscope. The control and HA groups were exposed to TSCI by dropping stainless-steel bars weighing 5 g from a 3-mm-wide and 10-cm-high tube, vertically, with the spinal cord exposed to 50 g/cm of impact. Damage to the spinal cord was created at approximately the level of thoracic 9 vertebra. Then, the skin incision was closed, step by step, in anatomical layers.

### 2.3. Blood sample analysis

Intracardiac blood samples collected from the rats were centrifuged at 1500 *g* for 10 min and the serum was separated and stored at −20 °C until analyzed. The serum total antioxidant status (TAS) and total oxidant status (TOS) levels were measured in the same autoanalyzer (AU5800, Beckman Coulter Inc., Brea, CA, USA) using commercial test kits (Rel Assay Diagnostics, Gaziantep, Turkey). Serum TAS and TOS were determined with kits (Rel Assay Diagnostics kit; Mega Tip) developed by Erel [8]. Oxidative Stress Index (OSI) values were also calculated.

### 2.4. TAS analysis

Serum TAS levels were determined using a novel automated measurement method, developed by Erel [8]. In this method, the antioxidative effect of the sample against the potent free radical reactions, which is initiated by the produced hydroxyl radical, is measured. The TAS results were expressed as mmol Trolox Eq/L.

### 2.5. TOS analysis

Serum TOS values were determined using a novel automated measurement method developed by Akbas et al. [9]. The color intensity, which can be measured spectrophotometrically, is related to the total amount of oxidant molecules present in the sample. The assay was calibrated with hydrogen peroxide (H_2_O_2_) and the results were expressed in terms of micro-molar H_2_O_2_ equivalent per liter (μmol H_2_O_2_ Eq/L) [10].

### 2.6. OSI value calculation

Oxidative stress index (OSI) values were calculated using the formula 100 × TOS (μmol H_2_O_2_ Eq/L) / TAS (mmol Trolox Eq/L). The results were expressed as arbitrary units (AUs).

### 2.7. Histopathological assessment

The pathological analysis was performed at the Department of Pathology of Tepecik Research and Training Hospital. Pathological specimens were collected after the rats were euthanized. Spinal cord samples were extracted from the trauma-affected region and placed into 10% formalin solution. The tissues were removed from the formalin solution, embedded in paraffin, sectioned at 3–4 μm, and stained with hematoxylin and eosin (H&E). An experienced pathologist examined the histological preparations with an Olympus BX51 light microscope (Olympus Scientific Solutions, Shinjuku, Tokyo, Japan), and photographs were taken with an Olympus DP72 camera (Olympus Scientific Solutions). In the tissue evaluations, changes in the severity of hemorrhage, edema, necrosis, polymorphonuclear leucocyte (PNL) infiltration, mononuclear leucocyte (MNL) infiltration, axonal swelling, and chromatolysis were investigated and scored, as follows: 0 = no visible change, 1 = minimal or slight change, 2 = moderate change, and 3 = severe change. At least 10 high-power fields (HPFs; 400X magnification) per section were examined for each sample.

### 2.8. Immunohistochemical TUNEL method

The terminal deoxynucleotidyl transferase dUTP nick end labeling (TUNEL) method, which detects the fragmentation of DNA in the nucleus during apoptotic cell death in situ, was employed using an In Situ Apoptosis Detection Kit (ApopTag; Merck Millipore, Burlington, MA, USA). All of the reagents listed below were from the kit and were prepared following the manufacturer’s instructions. However, the incubation times were increased. Xylene was used for deparaffinization of the sections and rehydrated through a graded ethanol series. The sections were then incubated with 20 μg/mL of proteinase K for 30 min at room temperature and rinsed in distilled water. Endogenous peroxidase activity was inhibited by incubation with 3% H_2_O_2_ in PBSs at room temperature for 15 min. The sections were then incubated with equilibration buffer for 3–5 min and then dUTP enzyme, in a humidified atmosphere at 37 °C, for 120 min. They were subsequently put into prewarmed working strength stop/wash buffer at room temperature for 10 min and incubated with antidigoxigenin conjugate for 60 min. Each step was separated by thorough washing in PBS. Labeling was revealed by applying peroxidase substrate, counter staining was performed using hematoxylin, and the sections were dehydrated, cleared, and mounted. To determine the TUNEL-positive cells, a HPF cell ratio was used as the index of apoptosis. To count the apoptotic cells, at least 10 microscopic HPFs in each section were evaluated and all of the positive staining nuclei in the field were counted. The TUNEL-positive nuclei were graded as follows: mild (1): 0–1, moderate (2): 2–9, severe (3): >10.

### 2.9. Neurological assessment

The Modified Tarlov Scoring system was applied for the motor function evaluation, which was evaluated at 24 h postop.

### 2.10. Statistical analysis

IBM SPSS Statistics for Windows 25.0 (IBM Corp., Armonk, NY, USA) was used to analyze the variables. The Kruskal–Wallis H test was used with the Monte Carlo simulation results in the comparison of the ordinal variables, which included the severity of hemorrhage, edema, necrosis, PNL infiltration, MNL infiltration, axonal swelling, chromatolysis, and paraplegia. The Dunn’s test was used for the post hoc analysis. Quantitative variables were grouped as the median, minimum, and maximum and presented in the Tables. The variables were examined at a 95% confidence interval (CI) and p < 0.05 was accepted as statistically significant.

## 3. Results

### 3.1. Neurological outcomes

No movement was observed in the control group, while an average of 0.25 and 1 point of motor function was observed in the HA 5 mg/kg and HA 10 mg/kg groups, respectively (Table 1). There was a significant statistical difference between the 4 groups (p < 0.001) (Table 1).

### 3.2. Histopathological and TUNEL staining

**Figure 1:**
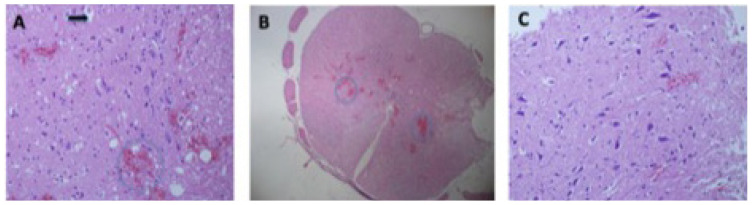
H&E staining showing: (A) dense hemorrhage areas (in circles), chromatolysis and inflammation (arrow) (200X) (control group); (B) hemorrhage spots at low magnification (40 X) (control group); (C) a sample with only minor areas of hemorrhage and low mononuclear lymphocytes (200 X) (HA 10 mg/kg).

**Figure 2:**
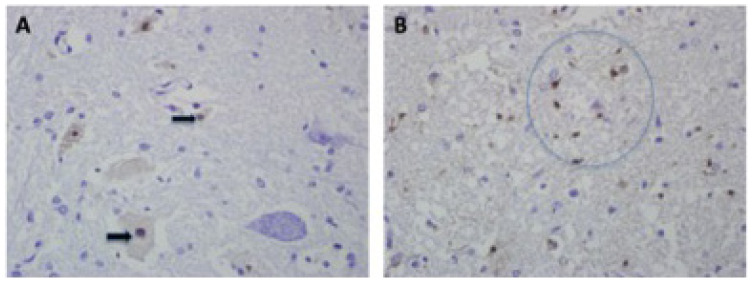
Positive staining (brown) with dUTP TUNEL in 2 different low-dose HA group specimens, showing apoptotic activity in the nucleus of the neurons (A) and wide range of cells (B).

When the groups were compared, there were significant dose-dependent changes in the severity of hemorrhage (p = 0.013), edema (p = 0.014), PNL infiltration (p = 0.018), and MNL infiltration (p = 0.019) in the HA 5 mg/kg and HA 10 mg/kg groups (Figures 1A–1C). However, no change was observed in the amount of axonal swelling (p = 0.39). Although a decrease of 0.5 U was found in the amount of chromatolysis, it was not statistically significant (p = 0.08) (Table 1). The number of TUNEL-stained neurons stained was lower in the sham group, as there was no TSCI. Moreover, there was no significant change in nuclei of the TUNEL-stained neurons when the control group was compared with the HA groups (p = 0.92) (Figures 2A and 2B)

### 3.3. Biochemical analysis

The preop blood samples showed no differences in the TAS, TOS, and OSI values. There was a significant decrease in the TAS values between the sham group and the HA 10 mg/kg group at 1 h postop. Although there was no significant difference in the TOS levels between the control and HA groups, there was a decrease in the TOS values at 1 h postop (p = 0.06). The OSI values were lower in the HA groups when compared to the control and sham groups, but the statistical analysis showed no significant result when they were analyzed together (p = 0.14)

There was a significant decrease in the TOS values in the HA 5 mg/kg group at 24 h postop compared to the control group. There was a decrease in the TOS values at 1 h postop in the HA 10 mg/kg group compared to the control group (p = 0.06) (Table 2). The OSI value at 24 h postop was lower in the HA 5 mg/kg group than in the HA 10 mg/kg group compared to the control group. However, this difference was not statistically significant.

## 4. Discussion

In the pathophysiology of TSCI, there are both primary and secondary injury cascades. The disintegration of ion hemostasis, glutamate excitotoxicity, mitochondrial dysfunction, and microvascular deterioration occur within secondary damage mechanisms and lead to oxidative stress by causing free radical formation directly or indirectly. As a result of uncontrolled chain reactions, secondary damage cascade further causes reactive oxygen species (ROS) production, inflammation, apoptosis, and neuronal damage. To date, no consensus exists regarding the agent to be used in TSCI, as there are harmful side effects.

One of the main concerns in TSCI is the ROS during the early stages. A sudden increase in superoxide and hydroxyl radicals (O2− and • OH) has been observed in TSCI models and it was stated that this increase remained high for up to 10 h [11,12]. An increase in malondialdehyde (MDA) was also observed in the first 5 h in lumbar puncture samples taken after TSCI in models [12]. Similarly, another study found an increase in MDA and cyclic guanosine monophosphate levels that lasted for about 1 h [12]. In TSCI rat models, using microdialysis and the high-pressure liquid chromatography, MDA has been shown to increase in as early as 2 h [12].

In the studies mentioned above, each of the oxidants and antioxidants were investigated separately. Herein, TAS and TOS analyses were performed. The TAS shows the body’s defense against oxidative stress. Antioxidants found in circulating blood aid in the removal of ROS. Antioxidants are transported through the blood to the whole body to maintain this redox balance. These redox reactions affect various antioxidants, thus it is not practical to measure all of them separately. It is more appropriate to measure the TAS. Likewise, the presence of endogenous oxidative enzymes such as xanthine oxidase, glycolate oxidase, and monoamine oxidase also make it beneficial to measure the TOS [13].

HA has been found to have antioxidant properties as a result of the phenol, carboxyl acid, and quinone in its structure [14,15]. Ozkan et al. [7] examined the antioxidant and oxidant effects of HA in pathological analyses in a cerebral ischemia model. They observed increased superoxide dismutase and nuclear respiratory factor-1 levels in the 10 mg/kg HA group compared to the control group, while the MDA levels were significantly decreased. Their histopathologic analyses revealed that less ischemia-related damage had occurred. Another study by Akbaş et al. [9] revealed a significant increase in the TAS values in a renal ischemia model. They reported that the TOS, OI, and ischemia modified albumin values were significantly lower in the HA groups compared with the control group. Tubular dilatation, tubular cell degeneration and necrosis, bowman capsule dilatation, tubular hyaline particles, and tubular cell distribution under H&E staining showed improvement compared to the control group. Apoptosis evaluation using the TUNEL technique indicated a significant decrease in apoptotic cells. Although there are examples of the benefits of HA in the literature, there are also articles presenting opposing views. Cheng et al. [16] reported an increase in superoxide anions and decrease in glutathione and other antioxidants with the administration of HA, resulting in oxidative stress.

In the current study, there were no significant differences between the HA groups in terms of the TAS values at 1 h postop. On the other hand, compared to the control group, the HA 5 mg/kg and HA 10 mg/kg groups showed a decrease in the TOS values at 1 h postop, but it was not statistically significant (p = 0.11 and p = 0.06, respectively). Although there was a decrease in the OSI values when comparing the control group with the HA 5 mg/kg and HA 10 mg/kg groups at 1 h postop, it was not statistically significant (p = 0.77 and p = 0.62, respectively). There was a statistically significant decrease in the TOS values in the HA 5 mg/kg group at 24 h postop (p < 0.05) (Table 2). According to these findings, with variable doses of HA after TSCI, there was no correlation between the improvement of movement and oxidative stress.

Moreover, the antiinflammatory effects of humic substances have been shown in previous studies either by blocking adhesion molecules or inhibiting the phagocytic stimulants [17]. It was found that humic substances cause antiinflammation by way of inhibiting the degranulation and adherence of neutrophils [17,18]. In another study by Goel et al. [18], it was reported that IP injection of HA had a strong antiinflammatory effect, causing a decrease in paw edema induced by protein injection in the legs of rats, which was used as a measure of inflammation. The relationship between HA and blood flow stimulating effects was also reported, which may be related to its antiinflammatory features [18,19].

In a study conducted by the European Agency for Evaluation of Medicinal Products, protective effects on the intestinal mucosa, and antitoxic and antimicrobial effects were also demonstrated [20]. Some long-term follow-up studies have shown several effects of HA. In the literature, the antiviral activity of HA was reported on human immunodeficiency virus in in vitro studies. Vuckits et al. [21] found increased humoral immunity response in HA-supplemented rats in a 26-day follow-up. They also observed an increased persistence of antibodies in the rat systems Joone et al. [22] revealed that oxyhumate increased interleukin-2 (IL-2) receptors and indirectly increased T-helper cell activity. Similar to the present study, but in a long-term follow-up (36 days), Weber et al. [23] reported that HA may play a role in negating the effects of oxidative stress, with no effect on the lipopolysaccharide induced IL-6 response. Çalışır et al. [24] studied the long-term effect of HA on wound healing. After 3 weeks of HA administration, they found a statistically significant difference between the saline control and the chlorhexidine gluconate group. They observed reduced inflammation and granulation tissue with a constricted mucosal epithelial layer.

In the current study, when the groups were evaluated together in terms of the pathological examinations, there was a statistically significant improvement in the severity of edema, hemorrhage, PNL infiltration, and MNL/microglia/macrophage infiltration (p < 0.05). In the paraplegia evaluation with the Modified Tarlov Scoring, there was a significant improvement in the HA groups compared to the control group (p < 0.001) (Table 1), which may have been related to the antiinflammatory or blood circulating effects of the HA.

This study did have some limitations. First, only the early stages of HA administration were studied, rather than the long-term effects. Second, other inflammatory pathways may be studied to reveal the possible effective cascade of HA. Finally, the groups comprised a low number of rats due to ethical issues.

Although no significant difference was found in the apoptotic cell count with TUNEL technique, there were significant histopathological changes, such as the decreased severity of hemorrhage, edema, PNL infiltration, and MNL infiltration. As the motor function was preserved significantly in the HA groups in a dose-dependent manner, these findings may be supportive of HA at different doses being effective on TSCI. This study investigated the early possible effects of HA in a TSCI model. The results must be further supported by larger case series, not only in the early stages but also over an extended period of time in chronic stages, as there may be different effects regarding the time and continuous dosing of the HA. The exact effect of HA on inflammatory substances and other possible mechanisms in TSCI should also be investigated so that it may be used as an alternative therapy in the future.

## Figures and Tables

**Table 1:** Statistical analysis of histopathological and neurological outcomes.

	Control group (I)	Sham group (II)	HA 5 mg/kg (III)	HA 10 mg/kg (IV)	p-value
	(n = 7)	(n = 7)	(n = 7)	(n = 7)	
	Grouped median (min/max)	Grouped median (min/max)	Grouped median (min/max)	Grouped median (min/max)	
Hemorrhage^a^	1.0 (0/2) ^II,III^	3.0 (1/4)	3.0 (2/4)	2.0 (1/3)	**0.013**
Edema^a^	0.5 (0/1) ^II,III^	1.3 (1/2)	1.1 (1/2)	1.0 (1/1)	**0.014**
Necrosis^a^	0.2 (0/1)	0.8 (0/1)	0.3 (0/2)	0.2 (0/1)	0.057
PNL infiltration^a^	0.0 (0/0)	0.8 (0/2) ^I,IV^	0.5 (0/2)	0.0 (0/0)	**0.018**
MNL infiltration^a^	0.3 (0/1) ^II,III^	1.3 (1/2)	1.3 (1/2)	1.0 (0/2)	**0.019**
Axonal swelling^a^	0.5 (0/1)	0.8 (0/1)	1.0 (0/2)	0.8 (0/1)	0.390
Chromatolysis^a^	0.3 (0/1)	1.2 (1/2)	1.0 (0/2)	0.5 (0/1)	0.080
Paraplegia^b^	4.5 (4/5) ^II,III^	0.2 (0/1) ^IV^	0.3 (0/1)	1.2 (1/2)	**<0.001**

a 0 = no damage, 1 = very mild, 2 = mild, 3 = moderate, 4 = severe;

b 0 = flasid, 1 = spastic, 2 = severe, 3 = moderate, 4 = mild, 5 = normal;

Kruskal–Wallis test (Monte Carlo), post hoc test: Dunn’s test.

**Table 2:** Statistical comparison of the biochemical findings between the groups (p-values).

	Control vs. HA 5 mg/kg	Control vs. HA 10 mg/kg	Sham vs. HA 5 mg/kg	Sham vs. HA 10 mg/kg	HA 5 mg/kg vs. HA 10 mg/kg	Control vs. Sham
TAS at 1 h postop	0.22	0.007	0.77	0.21	0.07	0.21
TOS at 1 h postop	0.096	0.07	0.11	**0.06**	**0.06**	0.77
OSI at 1 h postop	0.44	0.12	0.77	0.62	0.34	0.22
TAS at 24 h postop	0.71	0.09	0.88	0.74	0.61	0.93
TOS at 24 h postop	0.20	0.48	0.02	**0.06**	0.33	0.47
OSI at 24 h postop	0.56	0.07	0.83	0.24	0.17	0.52

Biochemical analysis of the TAS, TOS, and OSI values in the blood samples collected at 1 and 24 h postop (with either laminectomy or TSCI).
